# Possible role of ketone bodies in the generation of burst suppression electroencephalographic pattern

**DOI:** 10.3389/fnins.2022.1021035

**Published:** 2022-12-15

**Authors:** Dmitry Chegodaev, Vadim Gusev, Olga Lvova, Polina Pavlova

**Affiliations:** Ural Institute of Humanities, Department of Psychology, Ural Federal University, Yekaterinburg, Russia

**Keywords:** EEG, burst-suppression, mechanism, ketone bodies, metabolism

## Introduction

Burst-suppression (BS) is an electroencephalographic (EEG) pattern consisting of periods of high-amplitude slow and sharp waves, spikes (bursts) alternating with periods of background suppression up to the isoelectric line.

There are a considerable number of hypotheses proposing explanations for the genesis of BS. However, the most noteworthy are two: hypometabolism theory and hyperexcitability theory. From the viewpoint of hypometabolism theory, presented by the Ching et al. (2012), BS reflects a decrease in neural metabolism and the deficit of ATP is considered as a key event underlying of pathogenesis of BS. The second hypothesis considers BS as a result of cortical hyperexcitability, and the hyperexcitable state arises from the balance of excitation and inhibition being shifted toward excitation, implying the impairment of excitatory and inhibitory neurotransmitter systems (Mader et al., 2014; Shanker et al., 2021).

But several issues are related to BS, which are not accurately explained in terms of existing hypotheses. An important question is “why defects of corpus callosum are highly associated with disorders in which the BS can be observed?” Despite the fact that BS can be registered on EEG at all ages, it is highly specific for early infancy and especially for the neonatal period.

This paper discusses the potential relationship between the BS and brain ketone metabolism. We hypothesized that diminished brain's utilization of ketone bodies (KBs) is a key pathogenic factor, determining the appearance of BS on EEG. We base our speculative hypothesis on a brief overview of literature, provides circumstantial evidence for an important role of KBs in the pathogenesis of pathological conditions associated with BS. Taking our assumption into consideration, we attempted to shed light on the above-mentioned questions and clarify the possible mechanism underlying the BS.

## The link between the burst suppression pattern and malformations of corpus callosum: The view through the prism of ketone body metabolism

Notably, a BS is more frequently seen in disorders characterized by corpus callosum anomalies (Sharma and Prasad, 2017). Wide spectrum of genetic diseases including nonketotic hyperglycinemia, pyridox(am)ine-5-phosphate oxidase deficiency, pyridoxine dependent epilepsy, methylmalonic and propionic acidemia, maple syrup urine disease, sulfide oxidase deficiency, X-linked adrenoleukodystrophy (X-ALD), Ohtahara syndrome, 2-hydroxyglutaric aciduria demonstrates callosal abnormalities and burst-suppression pattern on EEG. Some arguments in favor of the involvement of KBs in the pathogenesis of these disorders can be briefly summarized as follows:

(1) Pyridoxine-dependent epilepsy, maple syrup urine disease, nonketotic hyperglycinemia, 2-hydroxyglutaric aciduria are characterized by disturbance in ketogenic amino acids (lysine, leucine, and glycine) metabolism. Ketogenic amino acids have been shown to provide a substantial proportion of KBs used for supply the immature brain. The pathways for KBs production from amino acids are apparently blocked in these diseases (Zinnanti et al., 2009; Rzem et al., 2015).
(2) Methylmalonic and propionic acidemia imply the inhibition of the utilization of acetoacetate and β-hydroxybutyrate into the brain, probably due to the competing propionate with ketones for the same membrane carrier and through inhibition of β-hydroxybutyrate dehydrogenase by methylmalonic acid (Dutra et al., 1991, 1993).
(3) β-oxidation defect in X-ALD is directly caused by ATP-binding cassette, subfamily d, member 1 (ABCD1) dysfunction. ABCD1 is a member of the ATP-binding cassette (ABC) transporter superfamily which is involved in the transport of very long chain fatty acids from the cytosol to the peroxisome. Therefore, the degradation of these fatty acids by peroxisomal β-oxidation is strongly reduced, which evidently leads in turn to the reduction of KBs production (Wiesinger et al., 2013; Berger et al., 2014).

The fact that KBs is highly important for myelination (Steiner, 2019) is the simplest and most plausible explanation for the link between disturbances of KBs metabolism and callosum abnormalities.

At least a few findings provide considerable support for this elucidation:

(1) During the early development of the brain, neurons and astrocytes were able to use KBs as a precursor for synthesis phosphatidylcholine, phosphatidylethanolamine and cholesterol (Poduslo and Miller, 1991), which are essential for myelin synthesis (Saher et al., 2005).
(2) Myelination is a critical process in the development of corpus callosum. Impairment of intrauterine myelination explains roughly the hypoplasia or thinning of corpus callosum (Nissenkorn et al., 2001; Andronikou et al., 2015).
(3) Agenesis of corpus callosum seems to depend primarily on the disturbance of cholesterol-dependent processes during embryological development that are responsible for impaired midline brain formation (Lee et al., 2013).

## Ketone bodies as a key determinant in the pathogenesis of pathological conditions that are typically associated with the burst suppression

The most common conditions linked to a BS also include profound general anesthesia, coma and hypothermia (Shanker et al., 2021). In order to provide a pathochemical basis for our hypothesis, we have also attempted to briefly highlight the participation of KBs in the pathogenesis of these conditions.

The leading cause of coma, which is more strongly associated with BS, is cardiac arrest (Sekar et al., 2019; Shanker et al., 2021). During severe heart failure, adaptive changes in cardiac metabolism greatly enhance the utilization of alternative energy substrates, such as KBs, explain well declines the KBs utilization in other tissues, including brain (Janardhan et al., 2011; Bernini et al., 2020).

Similar metabolic changes happen during hypothermia—another frequent etiological factor of BS. Enhanced secretion of glycocorticosteroids increases lipolysis and thus stimulate generation of KBs (Bańka et al., 2013). However, with continued hypothermia, further depletion of this energy resource will inevitably occur.

Several anesthetics, such as propofol and isoflurane, at high doses may produce BS (Shanker et al., 2021). A recent study (Stokes et al., 2021) shows that volatile anesthetics induce a dramatic depletion β-hydroxybutyrate. Propofol apparently inhibits carnitine palmitoyltransferase, which is critical for both astroglial and hepatic ketogenesis (Blázquez et al., 1998).

## How do ketone bodies modulate neuronal activity?

It is now evident that metabolic substrates also play a significant role in the modulation of neuronal excitability and could be considered a biochemical factor governing the genesis of different EEG patterns.

The ATP-sensitive potassium (KATP) channels are evidently primary candidates that provide coupling of metabolism and brain electrical activity. The assumption that KATP channels play a key role in coupling between KBs metabolism and neuronal excitability is supported by the finding that acetoacetate and β-hydroxybutyrate were found able to reduce the spontaneous firing rate of neurons, but this effect is not realized in the case of disruption of ATP-sensitive potassium channels (KATP) (Yellen, 2008). The importance of KATP in regulating neuronal activity is well illustrated by the finding that knockout of the pore-forming subunit Kir6.2 of KATP channels potentiates sensitivity to anoxia-induced seizures (Yamada et al., 2001) implying that KATP channels take part in preventing the excessive neuronal firing. This linkage between KATP and ketones, on the one hand, may be explained by the changing ATP cellular levels: decrease of ketone utilization by the brain lead to deficit in neuronal (global) ATP levels, and as a consequence of activation of KATP. This inference refers us to hypometabolism theory, which also implies the participation of KATP. On the other hand, the KBs apparently directly bind to (and activate) KATP channels, despite cellular ATP levels (Kim et al., 2015).

There are some other effects of KBs influencing neuronal activity, which are as follows:

(1) KBs can modulate the glutamate release. The possible mechanism could be that acetoacetate replaces Cl^−^, which leads to the inactivation of VGLUT, causing the suppression of vesicular glutamate release from neurons (Juge et al., 2010).
(2) KBs can decrease glycolysis, the central metabolic pathway that may promote seizure susceptibility, provide the energy required for epileptic activity during pathological conditions (Yang et al., 2013).
(3) Acetoacetate can enhance GABA synthesis in synaptosomes (Erecińska et al., 1996).

In sum, these findings show that KBs, even in physiological concentration, can serve as factors preventing the excessive excitatory activity. A deficit of ketones in the brain suggests the possibility of at least facilitation or/and initiation of the epileptic activity.

However, it is not sufficiently clear how KBs may contribute to the generation specifically of BS. Our explanation of this issue follows very closely the hypometabolism hypothesis, but it makes significant additions. The majority of pathological conditions with BS on EEG are characterized by deficit of intracellular ATP as a result of inhibition of oxidative phosphorylation and reduction in the use of as a fuel source [notably, that KBs can be used as a substrate for ATP production even during reduced oxygen availability (Kirsch and D'Alecy, 1984)]. However, ATP is necessary to supply the bursting activity. Apparently, anaerobic glycolysis (which is possibly facilitates under condition of the deficit of the KBs) is the primary metabolic pathway responsible for supplying the cells with ATP during bursts. It is important to note that glycolysis effectively produces ATP in the subplasmalemmal space, in close proximity to KATP, resulting in their inhibition (Tsuboi et al., 2004). Additionally, activity of the glycolytic enzymes and lack of direct activation by ketones are factors that can effectively inhibit KATP channels (Dhar-Chowdhury et al., 2005). The subsequent reduction of glycolysis leads to total deficit of cellular ATP (including subplasmalemmal ATP) and to the activation of KATP channels, which mediate membrane hyperpolarization during the suppressions.

## Discussion

In this short review we discussed the impact of KBs on the generation of epileptiform activity. Additionally, we suggested that the insufficiency of ketone bodies in the brain is critical for the appearance of BS on EEG. The assumption that there should be a specific substrate, whose depletion leads to subsequent deficiency of neuronal ATP not only does not contradict but also well complements prevailing hypotheses explaining the genesis of BS. However, so far, only indirect evidence supports our hypothesis and further studies are needed to clarify it.

## Author contributions

DC conceived of the presented idea and originated the concept and wrote the initial draft. VG, OL, and PP performed literature search and analysis contributed to the writing of the manuscript. DC and OL edited the manuscript. All authors provided critical feedback and helped shape the manuscript.
